# Fracture healing: cellular mechanisms and impact of parathyroid hormone and its analogs

**DOI:** 10.3389/fendo.2025.1703129

**Published:** 2025-11-17

**Authors:** Daniel Bikle

**Affiliations:** Departments of Medicine and Dermatology, University of California San Francisco and Veterans Administration Medical Center, San Francisco, CA, United States

**Keywords:** parathyroid hormone, fracture, skeletal stem cells, cellular signaling, abaloparatide, muscle stem cells

## Abstract

Fractures engender a multimillion dollar medical cost to society with substantial morbidity and mortality for the patients. In long bones fracture repair takes place at 3 distinct sites: intramembranous bone formation along the outer surface of the periosteum, endochondral bone formation bridging the fracture site, and intramedullary bone formation within the marrow at the ends of the fractured bone. Fracture repair occurs in 4 overlapping phases. 1. Hematoma formation and a proinflammatory response that activates the stem cells, 2. initiation of chondrogenesis, osteogenesis, and angiogenesis, 3. mineralization of the soft callus to form the hard callus, 4. remodeling of the callus to regenerate an intact bone. Stem cells in the periosteum, marrow, and overlying muscle supply the cells for the repair process. Parathyroid hormone (PTH) in its 1–34 form (teriparatide) and its analog abaloparatide are promising drugs to promote fracture repair. PTH acts via its receptor (PTHR) which is expressed in essentially all skeletal cells involved in fracture repair. Its anabolic actions are mediated by a number of interacting pathways including cAMP/PKA, Wnt, BMP, IGF1 and the bidirectional signaling of Ephrin B2/Eph B4. Progress in this field will lead to better treatment of fractures especially those slow or fail to heal.

## Highlights

1. Fractures result in major medical costs and substantial morbidity and mortality for the patients.
2. In long bones there are 3 distinct sites of fracture repair: intramembranous on outer side of the periosteum, endochondral bridging the fracture site, and intramedullary within the marrow at the ends of the fractured bones.
3. Fracture repair occurs in 4 phases starting with hematoma formation and inflammation activating the stem cell population to supply the cells needed to generate the callus, followed by mineralization of the callus which is then remodeled to the pre fracture state.
4. The stem cells responding to the fracture originate primarily from the periosteum, marrow, and overlying muscle although transdifferentiation of osteocytes at the fracture site contributes to intramedullary bone formation.
5. Parathyroid hormone and its analog abaloparatide are promising drugs to expedite fracture repair.

## Introduction: the clinical problem

In the United States there are an estimated two million fractures each year resulting in nearly a half million hospital admissions, 2.5 million office visits, and 180,000 nursing home admissions (1). Hip fractures account for only about 14% of all fractures, but they account for most of the costs and morbidity, estimated around $35,000 to $54,000 per fracture, with a 20-30% mortality within one year (2, 3). Although most fractures heal within 6–8 weeks, a significant percentage do not with delayed and even nonunion prolonging both the costs and morbidity (4). Bone turnover markers such as C-terminal telopeptide of collagen I (CTX), N-terminal propeptide of type 1 procollagen (P1NP) (5) or periostin (6) may provide markers for fracture healing. In the decades between 1970 and 2010, the rate of hip fractures in the Framingham Heart Study population decreased by 4.4%/year. This was attributed to better treatment of osteoporosis as well as a decrease in smoking and heavy drinking (7). However, this appears to have plateaued according to an analysis performed in 2015 (1). This likely relates to a decline in testing for osteoporosis and thus its treatment (8). Thus, fractures continue to be a major health problem. This review attempts to summarize what we know of the cellular mechanisms that are involved in fracture repair with a subsequent focus on how parathyroid hormone and its analogs impact those cellular mechanisms to promote the repair process. The discussion will be restricted to long bone fractures in which both intramembranous and endochondral bone formation occur as well as the less well studied intramedullary bone formation within the marrow at the ends of the fractured bone (9, 10).

## The three sites of fracture repair

**Figure 1:**
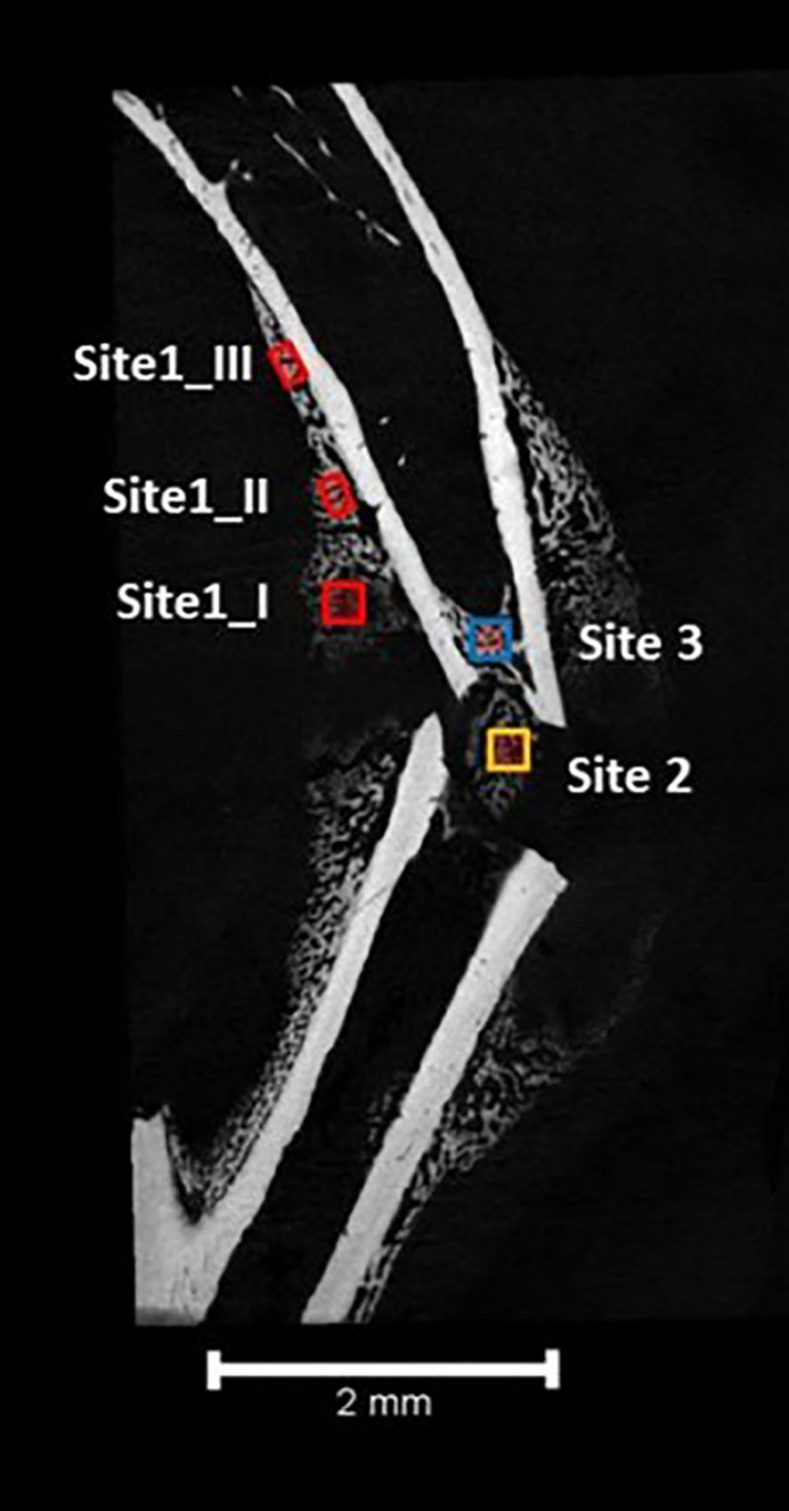
The three sites of long bone fracture repair. Site 1 involves intramembranous bone formation along the periosteum a short distance away from the fracture site. Stem cells from the periosteum as they differentiate into osteoblasts provide the source of progenitors for this site. Site 2 involves endochondral bone formation bridging the ends of the broken bone. Stem cells from the periosteum also provide the cells for this site as they differentiate first into chondrocytes. Site 3 occurs within the marrow at the ends of the broken bone. Stem cells from the marrow and the osteocytes within the cortical bone at the site of fracture differentiate or transdifferentiate into the osteoblasts for repair at this site.

The repair of fractures of long bones occurs in 3 sites of new bone formation (BF) that are distinct in both location and process as shown in **Figure 1** (9, 11). Site 1 marks the location of intramembranous BF along the periosteal surface of the intact bone. Bone formation at site 1 does not require an intermediate chondrocyte step. Site 3 marks the location of intramedullary BF forming within the marrow adjacent to the fracture site. BF at this site has been overlooked in studies using an intramedullary pin to stabilize the Fx as this obliterates the marrow space precluding new BF. As will be discussed BF at this site is quite distinct from BF at the other sites in the origin of the progenitor cells, although like that at site 1 a chondrogenic intermediary step is not involved. Site 2 is the bridging region between the broken ends of bone, and its development proceeds via endochondral BF that in many ways recapitulates the early development of bone (12).

## The four phases of fracture repair

**Figure 2:**
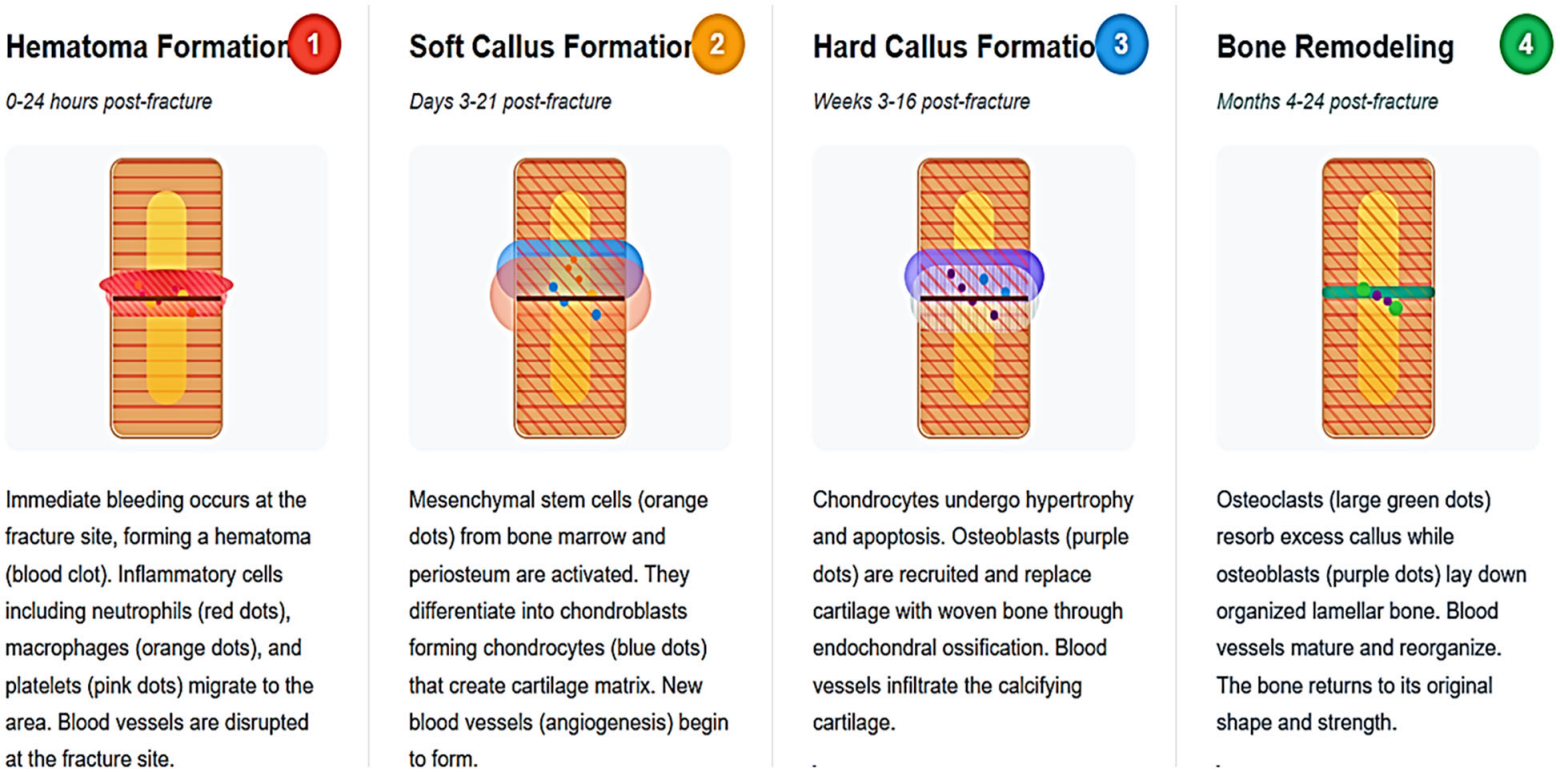
Four phases of fracture repair. The first phase involves formation of the hematoma into which inflammatory cells invade, activating the stem cells. The second phase includes angiogenesis and vascular invasion with the initiation of both endochondral bone formation to start forming the soft callus and osteogenesis in both the intramedullary and intramembranous sites. The third phase involves the mineralization of the soft callus with formation of the hard callus. The fourth phase marks the remodeling of the callus into lamellar bone to regenerate the bone to its pre fractured form.

The four phases of fracture repair have been well described in previous reports (13) and are summarized here and depicted in **Figure 2**. The repair process described is that of long bone fractures. This is a multifaceted process in which inflammation plays a key role. Factors such as age, comorbidities such as diabetes mellitus, obesity, and autoimmune diseases affect healing and contribute to the risk of dysunion or nonunion. That said in general phase 1 lasts about 5 days, phase 2 likewise about 5 days, phase 3 several weeks, and the final phase 4 months to even years (14) with overlap from one phase to the next.

### Phase 1

This phase is marked by the formation of a hematoma at the fracture site by erythrocytes and platelets infiltrated by immune cells from the marrow including neutrophils and monocytes (15, 16). The monocytes differentiate into macrophages that in the early pro-inflammatory environment develop the M1 phenotype that prefer the hypoxic environment of the initially avascular hematoma. They secrete a variety of inflammatory cytokines including chemokine (C-C motif) ligand 2 (CCL2) for neutrophil recruitment and granulocyte-colony stimulating factor (G-CSF) and granulocyte macrophage-stimulating factor (GM-CSF) for neutrophil maintenance as well as cytokines like the interleukins IL-1α and β, IL-2, IL-6, interferon gamma (IFNγ) and tumor necrosis factor alpha (TNFα) that activate T cells while recruiting mesenchymal skeletal progenitor cells (MSC) (17). (Skeletal stem cells, SSC, and mesenchymal stem cells, MSC will be used interchangeably as they are generally treated as such in the literature reviewed in this report). Moreover, the M1 macrophages induce receptor activator of nuclear factor kappa-b ligand (RANKL) expression in osteoblasts and T (in particular, Th17) cells to promote osteoclastogenesis necessary to initiate the remodeling of the callus (17). The apoptosis of the osteocytes at the fracture site further promotes the recruitment of osteoclasts (18). Following this initial proinflammatory response M2 macrophages appear by recruitment of macrophages from the bone marrow and transition of M1 to M2 macrophages. These cells secrete IL-10 to reduce the pro-inflammatory response (19), and transforming growth factor beta (TGFβ, vascular epithelial growth factor (VEGF), insulin like growth factor1 (IGF-1), and bone morphogenic protein 2 (BMP2) to promote vascularization and MSC differentiation into osteoblasts (20, 21). MSCs can likewise suppress the proinflammatory response by killing the inflammatory cells by direct contact and secreting cytokines such as TGFβ that induce Treg and inhibit neutrophil migration (22). Platelet derived growth factor (PDGF) secretion from macrophages stimulates periostin enabling the response of the periosteal stem cells to injury (23). Similarly platelets within the fracture callus also secrete PDGF, VEGF, TGFβ, and IGF-1 facilitating the increase in vascularization, proliferation of MSCs, osteoblast differentiation and chondrogenesis (24).

### Phase 2

This phase is marked by angiogenesis, cartilage formation, and development of the soft callus. Endochondral bone formation occurs in the region between the fractured ends of the bone, the bridge region, while intramembranous bone formation occurs along the periosteum at some distance from the fracture site. A third site, that within the medullary regions at the ends of the fractured bone, is best seen in non-stabilized fractures or in drill hole models. Intramedullary bone begins somewhat later (day 5–10 in mice) and is transient (9–11). At the first two sites stem cells from the periosteum proliferate and differentiate into osteoblasts or chondrocytes (25, 26), although stem cells from muscle also contribute (27–29). Runx2 regulates osteoblastogenesis, whereas osterix suppresses runx2 facilitating the development of chondrocytes (30). Preceding the expression of runx2 is upregulation of the glucose transporter GLUT 1, indicating the high energy demands for osteoblastogenesis (31). Hedgehog signaling interacting with both the wnt and BMP pathways regulates these early events in osteoblast differentiation and endochondral ossification (32). Notch signaling inhibits wnt signaling leading to proliferation of osteoprogenitors but inhibition of their differentiation (33), whereas BMP (in particular BMP2) signaling promotes osteoblast differentiation (34). IGF-1 promotes both chondrocyte and osteoblast proliferation and differentiation (35) and is a key mediator of the role of parathyroid hormone (PTH) in this regard (36). Ephrin B2 plays a critical role in coordinating these events (11, 37) as does calcium via its calcium sensing receptor (38). Intramedullary bone formation appears to be initiated following transdifferentiation of osteocytes from the cortex at the fracture site (9, 39) and differentiation of leptin receptor (LepR+) expressing stem cells in the marrow (40). The cartilage collar forms the soft callus starting at day 3 in mice, but it subsequently mineralizes, with the chondrocytes initially becoming hypertrophic, switching from producing collagen II to collagen X and eventually transdifferentiating into osteoblasts, a process reminiscent of that in the growth plate (41). This resemblance to bone formation at the growth plate is further supported by a genetic analysis of the genes involved showing similarity between fracture healing and embryonic development of bone (42). IL-6 plays an important role in the mineralization process (43). Vascularization of the soft callus is driven primarily by VEGF, fibroblast growth factor 1 (FGF1), and TGFβ coming from the M2 macrophages and platelets as noted above as well as osteoblasts (44). The type of vessel may also matter. Endomycin expressing endothelial cells (H cells) are more associated with osteoprogenitors and osteogenesis than endomycin low endothelium (45). Intramembranous bone formation along the periosteum develops without going through a chondrocyte phase to form the hard callus. Intramedullary bone formation likewise does not proceed through a chondrocyte intermediate step. Unlike the development of the soft and hard callus, it begins between 5–10 days, and is essentially gone by 28 days (9, 10).

### Phase 3

This phase is marked by cartilage removal and its calcification with more complete formation of the hard callus. The chondrocytes mineralize and are either removed by osteoclasts or transition into osteoblasts (9, 46, 47). This process is accompanied by and perhaps induced by vascularization as the hypertrophic chondrocytes lose their sox9 and collagen X expression while increasing runx2 and β-catenin expression followed by that of osteoblast markers such as alkaline phosphatase, osterix, osteopontin, and osteocalcin (13, 48). Tumor necrosis factor alpha (TNFα) plays a role in stimulating osteoclast formation, and likely plays a role in the mineralization process (49). The induction of matrix metalloproteinases such as MMP13 facilitates the degradation of the matrix (47). The down regulation of noggin, an inhibitor of BMP stimulation of osteoblast differentiation, is also required for the mineralization process to proceed (50). Woven bone is initially formed that with the recruitment of osteoclasts is remodeled into lamellar bone during the fourth phase.

### Phase 4

This phase involves the remodeling of the woven bone into lamellar bone to restore the bone to its pre fracture condition including restoration of the hematopoietic and trabecular structures. This process begins after 3–4 weeks but extends for months. Cytokines such as IL-1, IL-6 and TNFα are major drivers (28, 47, 51). BMPs promote cartilage resorption and recruitment of osteoblasts (51, 52) with the wnt signaling pathway playing a major role (18). The cells involved include the osteoclasts that resorb the cartilage and help remodel the woven bone with subsequent rebuilding of the lamellar bone by osteoblasts (47, 53, 54). With time the number and role of osteoclasts diminishes as osteoblasts and osteocytes become the primary bone cells, and in human bone the Haversian system with its central vascular canal and canalicular network are reestablished (55). Human osteoblasts from fractures that fail to heal show marked changes in these pathways of growth and differentiation (56).

## Skeletal stem/progenitor cells during fracture repair

**Figure 3:**
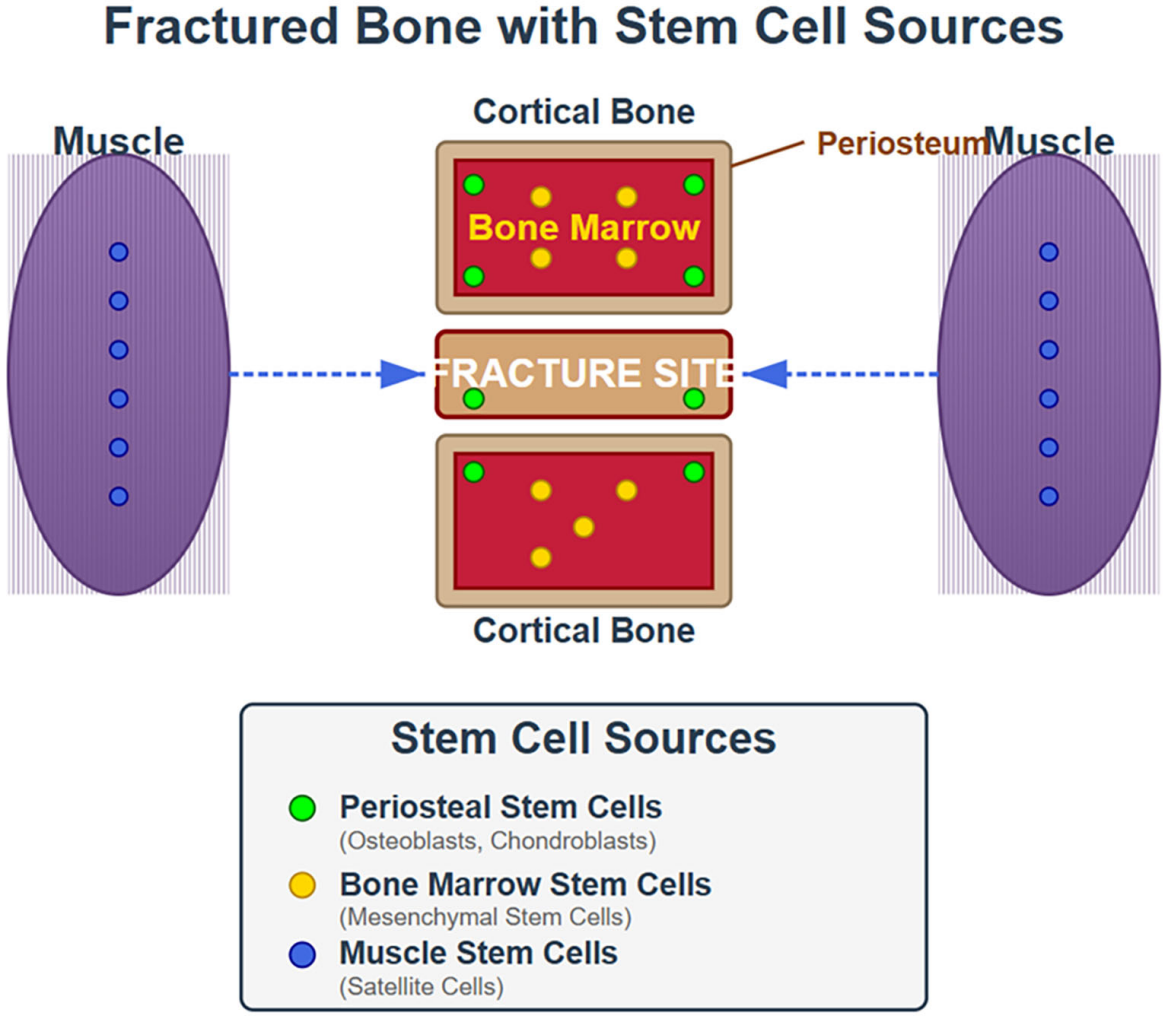
Origin of stem cells involved in fracture repair. This cartoon illustrates that during fracture repair stem cells from the periosteum, bone marrow and muscle all contribute albeit to different extents.

In an initial quest to identify the skeletal stem cell from which all skeletal progenitor cells were derived Chan et al (57) identified cells from the mouse growth plate that they purified by fluorescence activated cell sorting (FACS) that were capable of differentiating into bone, cartilage, and stromal cell lineages. These cells, called osteochondral skeletal stem cells (ocSSC), were identified with the markers CD45^-^,Ter119^-^,Tie2^-^,αυ^+^, Thy^-^,6C3^-^,CD105^-^,CD200^+^. The authors hypothesized that this cell type was the source of all the osteochondroprogenitors similar to the single stem cell in the hematopoietic system leading to the subsequent cell lines. Similar cells were found in the fracture callus, which the authors called fracture induced bone, cartilage, stromal progenitors (f-BCSP) (58). Several years later Chan et al. identified what they considered the human skeletal stem cell in this case marked as PDPN^+^,CD145^-^,CD73^+^,CD164^+^ which like the mouse skeletal stem cell could be induced to produce bone, cartilage, and stromal cell lineages. These cells were not progenitors for fat or muscle. However, this group subsequently identified a second group of stem cells called perivascular skeletal stem cells (pvSSC) identified with markers CD45^-^,CD31^-^,Pdgfrα+,Sca1^+^,CD24^+^. These cells are the source of adipogenic progenitors. but also osteochondrostromal progenitors (59). These cells met the stem cell criteria of being self-renewing and multipotent. Furthermore, these cells were induced to proliferate in response to injury such as fracture, and produce the more differentiated cell lines required to heal the fracture. The concept of a single skeletal stem cell was further challenged by the discovery of other niches of skeletal stem cells in different regions of the skeleton and under different regulation (25, 40, 60–62). Moreover other tissues like fat and muscle could provide stem cells that contributed to fracture repair (40). The main niches for skeletal stem cells involved in fracture repair of long bones are the periosteum, marrow, and muscle (**Figure 3**).

### Periosteal stem cells

These cells reside in the inner cambium layer of the periosteum (63). They are considered the main source of cells involved in fracture repair of long bones (27) and are superior to bone marrow stem cells when used in therapy for fracture healing (64). In the mouse they express markers such as CD90, CD105, CD51, and CD29, Sca1 and in humans CD90, CD105, and CD73 (65). In response to injury (fracture) these stem cells lose their markers and change their cell fate, for example from generating only osteoblasts within the kidney capsule to generating both chondrocytes and osteoblasts in response to fracture (66). To trace the fate of these cells and their progeny in response to fracture various cre recombinases (cre) have been used to label the cells in combination with a fluorescent probe activated by the cre. These cres have not proved to be highly specific. However, gli 1 cre is a useful means of labeling periosteal stem cells and their response to injury (67) in contradistinction to adiponectin cre which preferentially labels Leptin receptor + (LepR^+^) cells in the bone marrow. The glioma associated protein (gli1+) expressing stem cells give rise to periostin (postn) + cells (68), many of which express the markers of the stem cells identified by Chan et al (57). Cathepsin K (ctsk) cre, alpha smooth muscle actin (αSMA) cre, and paired homeobox 1 (prrx1) cre have also been used to label periosteal stem cells, but also label bone marrow stromal cells and other tissues (67, 69). These cres do not necessarily label the same cells, as cells from the periosteum with characteristics of skeletal stem cells have been shown by scRNA-seq to be heterogeneous (63, 70). The review by Salhotra et al (32) provides a good overview of the various transcription factors and pathways involved in the fracture repair process as the stem cells differentiate into their different fates.

### Bone marrow stem cells

In the adult LepR^+^ expressing SSC are a major source of osteoblasts (59). These reside as perivascular stromal cells in the bone marrow. These cells largely overlap with CXCL12^+^ abundant reticular cells (CAR cells) in the marrow, and both contribute to either osteogenesis (osteo-CAR) or adipogenesis (adipo-CAR) subsets (71, 72).The osteo-CAR cells are associated the endomucin high expressing H type vessels mentioned earlier, whereas the adipo-CAR cells are associated with endomucin low expressing L type vessels (sinusoid) or non vasculature (40). Zinc finger protein 467 appears to regulate the differentiation of these stem cells promoting the adipogenic fate (73, 74). The osteo-CAR cells contribute to fracture repair (75), but the role of the adipo-CAR cells is less clear. However, LepR^+^ Adiponectin^+^ cells, likely overlapping if not the same as adipo-CAR cells, were the major contributors to bone formation within drill hole injuries (67) and may be a source of cells forming the intramedullary bone during bicortical fractures (9). Similarly marrow adipogenic lineage precursors also likely overlap adipo-CAR cells, and have been shown to promote osteoclastogenesis and secrete BMP receptor inhibitors to inhibit bone formation during skeletal remodeling (76, 77). Their role during fracture repair is not clear. The interferon-induced GTP binding protein Mx1 marks an osteoprogenitor pool of cells on the endosteal surface and bone marrow that contributes to fracture repair (78). Other markers have labeled various cells within the bone marrow, but they also label periosteal stem cells so determining their distinct contribution to fracture repair is unclear.

### Muscle stem cells

Although not strictly speaking skeletal stem cells, the juxtaposition of muscle to bone, and the injury to both during fractures has generated interest in the contribution of muscle to fracture repair. Separating muscle from bone over fracture sites with a cell impermeable membrane disrupts the repair process (79). Moreover, muscle secretes a number of myokines that promote fracture repair (80). Fibro-adipogenic progenitors (FAP) cells in muscle are key cells involved in muscle regeneration (81). But they also have osteogenic potential (80). Julien et al (27) labeled muscle stem cells with prrx1 cre and demonstrated their incorporation into the skeletal repair process. The Pax7 or Pax3 muscle satellite cells do not appear to play a role (82). He et al. (83) labeled Prg (lubricin) expressing FAP cells and likewise showed their contribution to chondrocytes and osteoblasts during fracture repair. Similar results were observed when muscle cells were labeled with αSMA cre, that labeled both perivascular and satellite cells, although Pax7 labeled satellite cells did not contribute (84).

## Role of parathyroid hormone and its analogs in fracture repair

### Clinical and animal studies

Parathyroid hormone (PTH), in particular the 1–34 portion of PTH (teriparatide), is effective in promoting fracture repair in a number of animal and human studies (85–87), although it is not yet FDA approved for this purpose in humans. Gorter et al. (88) summarized 41 animal studies in which various drugs, antiresorptive and anabolic, were used to treat fractures in either osteoporotic or non-osteoporotic animal models. PTH proved superior to the antiresorptives such as alendronate in showing an increase in bone mineral content, callus formation, biomechanical strength, improved radiologic and histologic healing, and improved union rate (89–91). Clinical studies have been less consistent. Retrospective studies (92, 93) and some randomized controlled trials (RCT) (94–96) but not all RCT (95, 97) have shown acceleration of union, although the incidence of non unions does not appear to be reduced.

Abaloparatide (Abl) is a synthetic 36aa analog of parathyroid hormone related peptide (PTHrP) to which it shares 76% homology and 42% homology with teriparatide (98). It signals through the same receptor as PTH and PTHrP and also promotes fracture repair (89). Although both teriparatide and abaloparatide act via the same receptor, their effects on the receptor differ (99). The parathyroid receptor has two conformations: R° and R^G^. Abaloparatide has enhanced selectivity for the R^G^ form of the receptor relative to the R° form compared to teriparatide. The R^G^ conformation mediates a faster and less sustained cell signaling response associated with a greater anabolic and less catabolic effect on bone metabolism (100). The molecular basis for this is well described in a review by Vilardaga et al (101). These differences also lead to substantial differences in their effects on the transcriptome (102). Several animal studies comparing abaloparatide to teriparatide on the balance between bone formation and bone resorption have demonstrated a greater increase in the expression of genes associated with anabolism and bone mineral density with abaloparatide (103, 104). For example, abaloparatide and teriparatide differ in their modulation of RANKL expression (abaloparatide has a lesser effect) perhaps explaining part of their differences in their effects on fracture repair (100, 105). In animal studies lower doses of abaloparatide have a greater effect on trabecular and cortical bone than equivalent doses of teriparatide (106, 107), although at higher doses the effects on bone of the two drugs appear to be equivalent (103). However, in a study in ovariectomized monkeys abaloparatide did not increase cortical porosity or elevate serum calcium (108), unlike earlier studies with teriparatide or full length PTH in this animal model (109, 110), indicating a lesser effect on bone resorption. With respect to clinical studies the Dose-finding Study of Abaloparatide (ACTIVE trial) demonstrated that 40 and 80ug abaloparatide increased bone mineral density at the lumbar spine, femoral neck, and total hip vs placebo and more than a 20ug teriparatide dose at the hip. This occurred with less increase in the bone resorption marker CTX-1 and fewer incidences of hypercalcemia (111, 112). Moreover, in a meta-analysis abaloparatide showed a greater reduction in both vertebral and non-vertebral fractures compared to teriparatide (113).

### Cellular mechanisms of action during fracture repair

PTH originates only from the parathyroid gland, whereas PTHrP is expressed in a number of tissues including the periosteum of bone, where its expression is increased following fracture. Mice deficient in either PTH or PTHrP have delayed fracture repair (114, 115) including deletion of PTHrP specifically from the periosteum (116).The PTH/PTHrP receptor is found in a number of bone cells that participate in fracture repair including mesenchymal stem cells (MSC) in the marrow and periosteum, chondrocytes, osteoblasts, and osteocytes (117), and thus would be expected to participate in all sites of bone formation during fracture repair. In the marrow PTH/PTHrP likely promotes intramedullary bone in part by downregulation of the transcription factor Zfp467, that otherwise promotes adipogenesis while blocking osteogenesis (74, 118). The ability of PTH and PTHrP to stimulate the proliferation of osteochondral progenitors (119), and act on both chondrocytes and osteoblasts to promote chondrogenesis and osteogenesis (85, 120, 121), is critical to fracture repair. Runx 1 appears to play a major role in PTH stimulated chondrogenesis (122), whereas a number of transcription factors including runx2 play a role in osteogenesis. PTH also promotes angiogenesis (123), critical for remodeling during fracture repair (124). This includes not only the vascular invasion during the endochondral phase of bone formation, but the increase in blood vessels within the marrow and the bone cortex itself (123–125). In these actions PTH promotes the proliferation of the αSMA^+^ perivascular stromal cells (126). As will be discussed subsequently, PTH not only increases the cross talk between chondrocytes, osteoblasts and osteoclasts via ephrinB2/EphB4 signaling (37, 127) but also the cross talk between ephrinB2 expressing osteoblasts and the EphB4 expressing endomucin+ blood vessels in bone (123). The increase in intracortical and intramedullary blood vessels could provide perivascular osteoprogenitors for intramedullary bone formation during fracture repair as noted above.

### PTH acts through a number of different pathways

These have been well reviewed by Liu et al (128). The PTH receptor activation of the cAMP/PKA and the Ca^2+^/PKC pathways occurs via distinct G proteins, G_s_ and G_q_. G_12/13_ mediates the phospholipase D-transforming protein RhoA pathway that has been little studied in fracture repair. In general the anabolic pro osteogenic actions are mediated through the cAMP/PKA pathway (129, 130), whereas the Ca^2+^/PKC pathway underlies the catabolic actions (131). In skeletal stem cells (SSC) the increase in cAMP leading to the increase in PKA activity and the cAMP response element binding protein (p-CREB) promotes their proliferation and osteogenic differentiation (130). Although the Ca^2+^/PKC pathway has been associated with catabolic actions, at least one report indicates that PKC_δ_ may promote osteogenesis in MSC (132). The PKA-SIK (salt inducible kinase) pathway plays an important role downstream of PTH activation of the cAMP/PKA pathway. PTH by activating PKA phosphorylates and inactivates SIK2 which in the non-phosphorylated form would phosphorylate the histone deacetylases HDAC4 and 5 keeping them out of the nucleus. However, with SIK2 inactivated, HDAC 4 and 5 can enter the nucleus where they inhibit the function of MEF2, a transcription factor that otherwise drives Sost expression, an inhibitor of bone formation (133). Inactivation of SIK2 also enables the CREB regulated transcription factor (CRTC2) to enter the nucleus promoting bone resorption (133). The transcription factor nascent polypeptide-associated complex and coactivator alpha (αNAC) is also a substrate for PTH activated PKA. Its phosphorylation enables its entrance into the nucleus where it promotes expression of genes involved with bone formation (134). αNAC also colocalizes with and facilitates junD stimulation of LRP6 expression (135). As described below LRP6 serves as a coreceptor for the PTH receptor (PTHR) enhancing its function.

Wnt pathway. PTH stimulates the Wnt pathway thus increasing active β-catenin levels in periosteal SSC expanding this population to provide the cells for fracture repair (136). PTH increases the levels of other members of the Wnt signaling pathway including LRP5, Wnt7b and Wnt10b (137) while reducing Wnt inhibitors such as Dkk1 (138), Wasf2 (139), and in osteocytes Sost (140). At least some of the Wnt10b comes from PTH stimulation of T cells (141). The coreceptor for the Wnt receptor frizzled (FZD), LRP6, also serves as a coreceptor for the PTH receptor (PTHR) facilitating PTH stimulation of cAMP production (142). The enhanced PTHR/LRP6 signaling in turn can inhibit sclerostin production via the PKA/SIK2 pathway feeding back positively on Wnt signaling including PTHR/LRP6 signaling, a feed forward loop. However, this interaction is blocked by N-cadherin providing some negative control (143). The PTHR/LRP6 signaling pathway in osteocytes could contribute to PTH promotion of intramedullary bone formation during fracture repair but has not been tested.

BMP/SMAD pathway. PTH activates the SMAD1 pathway in the periosteum, mediating downstream targets of BMP2 and increasing osteogenesis (144). This is activated by the PTH induced endocytosis of the PTHR/LRP6 complex leading to phosphorylation of SMAD1 and stimulation of αSMA^+^ stem cell differentiation into osteoblasts (145).

IGF1 signaling. PTH stimulates the expression of IGF1 and its receptor (IGF1R) in bone (146, 147). IGF1 (148) and its receptor (149) are required for the anabolic actions of PTH on bone. The chondrocytes in the growth plates (GP) of global ^(glo)^IGF1KO mice show decreased proliferation and increased apoptosis. Differentiation of the proliferating chondrocytes to hypertrophic chondrocytes is delayed with decreased vascular invasion and mineralization (150). The delay in vascular invasion is consistent with the induction by IGF1 of hypoxia inducible factor (HIF)1α and vascular endothelial growth factor (VEGF) (151). Bone formation is reduced in the ^glo^IGF1KO mice that survive postnatally. Although bone formation is reduced, trabecular bone volume (BV/TV) in the proximal tibia is increased (152), a result reflecting the dual effect of IGF1 on osteoblast and osteoclast activity (153, 154). IGF1 stimulates RANKL expression by osteoblasts thus promoting osteoclastogenesis (154–156). IGF1 signaling plays a comparable critical role during fracture repair. IGF1 and IGF1R are expressed in chondrocytes, osteoblasts, and osteoclasts, and their levels increase throughout fracture repair (157, 158). This is associated with a marked increase in proliferation of cells within the cambial layer of the periosteum, increase in Sox2 labeled stem cells, increase in VEGF expression, and invasion into the callus. Shi et al (159) demonstrated that PTH stimulated gli1^+^ stem cell progenitor differentiation in this case to chondrocytes via IGF signaling, and it is likely that IGF signaling underlies other PTH actions during fracture repair.

Ephrinb2/EphB4 signaling. Among the means by which PTH and IGF1 promote the intercellular communication required for fracture repair involves the bidirectional ephrinB2 (EfnB2)/EphB4 signaling, which when disrupted impairs fracture repair (11). This bidirectional signaling of EfnB2/EphB4 occurs between all cells in bone (127) including the blood vessels (123, 160). Of particular relevance to fracture repair is that this bidirectional signaling promotes the motility of mesenchymal stem and endothelial cells, promotes osteochondral differentiation, and stimulates VEGF induced angiogenesis (161–164). In fracture studies with ^col2^EfnB2KO mice (11) vascular invasion was markedly reduced as were VEGF expression and numbers of stem cells in the invasion front. IGF1 is essential for the expression of EfnB2 and EphB4 in those cells engaged in fracture repair (37). Stimulation of EfnB2 and EphB4 expression by PTH (165) requires IGF1/IGF1R. Blocking the EfnB2/EphB4 interactions with specific inhibitors or gene deletions blocks basal and IGF1 stimulated chondrocyte, osteoblast, and osteoclast differentiation, and blocks IGF1 stimulated fracture repair (11). Thus, like PTH and IGF1, EfnB2/EphB4 signaling is found in and between essentially all the cells involved with fracture repair and is required for the fracture repair process.

## Conclusions

Fractures are a major medical concern, and especially if the repair process is prolonged, leading to huge morbidity, mortality, and costs. Thus understanding the mechanisms by which fracture repair takes place will likely lead to better therapy improving the outcomes for the patients involved. Repair of the fracture rallies all cells in the bone and bone marrow to alter their cell fate with the goal of regenerating bone to its pre fracture condition. Within bone and bone marrow exist different stem cell niches. It is these cells that respond to the challenge to alter their cell fate to repair the fracture. Indeed, these skeletal stem cells are being used clinically to expedite fracture repair especially in conditions of delayed repair or nonunion. One class of drugs, PTH and its analogs also shows promise in facilitating the repair process. As is appropriate for the complexity of fracture repair, a number of signaling pathways are activated by PTH to promote fracture repair. Many of the anabolic actions of PTH are mediated by the cAMP/PKA pathway. However, Wnt signaling, IGF1 signaling and the bidirectional signaling of EfnB2/EphB4 play major and interacting roles as well. With progress in this field we can anticipate that the burden of fractures, especially those with delayed or non-unions will be lessened.
